# Fumarase activity: an *in vivo* and *in vitro* biomarker for acute kidney injury

**DOI:** 10.1038/srep40812

**Published:** 2017-01-17

**Authors:** Per Mose Nielsen, Abubakr Eldirdiri, Lotte Bonde Bertelsen, Hans Stødkilde Jørgensen, Jan Henrik Ardenkjaer-Larsen, Christoffer Laustsen

**Affiliations:** 1 MR Research Centre, Department of Clinical Medicine, Aarhus University, Aarhus, Denmark; 2 Department of Electrical Engineering, Technical University of Denmark, Kgs Lyngby, Denmark; 3 GE Healthcare, Broendby, Denmark

## Abstract

Renal ischemia/reperfusion injury (IRI) is a leading cause of acute kidney injury (AKI), and at present, there is a lack of reliable biomarkers that can diagnose AKI and measure early progression because the commonly used methods cannot evaluate single-kidney IRI. Hyperpolarized [1,4-^13^C_2_]fumarate conversion to [1,4-^13^C_2_]malate by fumarase has been proposed as a measure of necrosis in rat tumor models and in chemically induced AKI rats. Here we show that the degradation of cell membranes in connection with necrosis leads to elevated fumarase activity in plasma and urine and secondly that hyperpolarized [1,4-^13^C_2_]malate production 24 h after reperfusion correlates with renal necrosis in a 40-min unilateral ischemic rat model. Fumarase activity screening on bio-fluids can detect injury severity, in bilateral as well as unilateral AKI models, differentiating moderate and severe AKI as well as short- and long-term AKI. Furthermore after verification of renal injury by bio-fluid analysis the precise injury location can be monitored by *in vivo* measurements of the fumarase activity non-invasively by hyperpolarized [1,4-^13^C]fumarate MR imaging. The combined *in vitro* and *in vivo* biomarker of AKI responds to the essential requirements for a new reliable biomarker of AKI.

Acute kidney injury (AKI)^1^,^2^,^3^ occurs in 1.9% of all hospital in-patients^4^. The illness is especially common in critically ill patients, and the prevalence in this group is >40% at admission to the intensive-care unit if sepsis is present^4^. The underlying causes of AKI include sepsis, toxins, and urethral obstruction. However, the main contributor is renal ischemia/reperfusion injury (IRI), which accounts for up to 47% of all cases of AKI^2^. IRI can be caused by kidney transplants, hypovolemia, cardiogenic shock, and renal vascular diseases^2^,^5^. The effective treatment of AKI should begin at the earliest sign of renal dysfunction, but the current preferred biomarkers of AKI such as plasma creatinine, creatinine clearance (CrCl), glomerular filtration rate (GFR) determined using Inulin or Cr-EDTA^6^,^7^ and blood urea nitrogen (BUN)^8^,^9^ lack specificity and sensitivity as they only rise substantially above the normal levels once renal damage has already occurred.

The parameters listed above reflect the residual glomerular filtration rate rather than injury itself^10^. Alternatively, renal biopsies can identify single-kidney or local IRI by measuring the protein or mRNA expression levels of lactate dehydrogenase, kidney injury molecule 1 (KIM-1), and neutrophil gelatinase-associated lipocalin (NGAL)^11^. Renal biopsies provide high sensitivity, but are associated with the high risk of additional chronic injury and hemorrhage that should ideally be avoided in critically ill patients^12^,^13^. Thus, precise and non-invasive methods, preferably imaging methods or the sampling of urine and blood to continuously and directly evaluate the severity of single-kidney IRI in patients and animals, are urgently needed.

Hyperpolarization of ^13^C-labeled molecules leads to a >10,000-fold increase in signal compared to conventional magnetic resonance imaging (MRI)^14^. This signal enhancement allows real-time imaging of metabolic pathways using ^13^C-labeled endogenous substrates^15^. We have recently demonstrated metabolic alterations in post-ischemic unilateral IRI rats following hyperpolarized [1-^13^C]pyruvate infusion^16^. Showing an upregulation of the anaerobic pathways, similarly to what has been demonstrated in diabetic kidney^17^,^18^,^19^, albeit lower total turnover, most likely caused by necrosis. In principle any small molecular probe can be hyperpolarized as long as they contain a nuclear spin, typically ^13^C^20^,^21^. Currently there is an large array of commercial available hyperpolarized of ^13^C-labeled molecules which have been utilized in many different animal models of cancer^22^,^23^, myocardial ischemia^24^,^25^,^26^ and renal diseases^27^,^28^,^29^,^30^, and more recently in patients and healthy volunteers^31^,^32^ Additionally, [1,4-^13^C]fumarate has been shown to be a reliable imaging biomarker for monitoring cellular necrosis^33^ in a rat model of xenograft tumors and subsequently in a rat model with folic acid-induced AKI^34^. These previous results showed an increased ^13^C-malate signal in kidneys with progressive tubular necrosis. In intact cells, the uptake and subsequent hydration of fumarate to malate occurs too slowly compared to the hyperpolarization signal decay, whereas in necrotic cells the cellular integrity is broken, allowing fumarate to enter the cell and fumarase to leak out. From this point, the enzymatic conversion via fumarase occurs rapidly and the malate product is detectable by MRI. This might also cause the release of fumarate in the urine and blood, yielding a biomarker detectable in the plasma and urine.

Based on the above, we question if hyperpolarized [1,4-^13^C]fumarate can be used to asses renal necrosis in connection with IRI, and will be associated with fumarase activity in both urine and blood.

## Results

**Figure 1:**
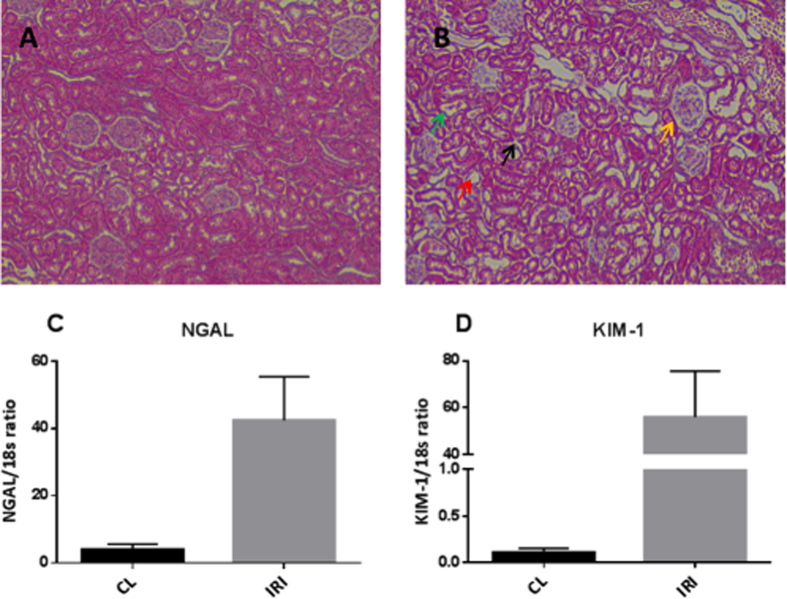
Verification of ischemia-reperfusion injury. Representative histological sections are shown in (**A**) a CL kidney showing normal intact tubular cells and glomeruli, and (**B**) a post-ischemic kidney showing a cellular cast in the tubular lumina (green arrow), complete sloughing of tubular epithelium (red arrow), interstitial edema (black arrow), and glomerular edema (yellow arrow). Magnification 20×, HE stain. The relative expression of injury markers indicated significant upregulation of (**A**) NGAL (p = 0.0145, n = 6) and (**B**) KIM-1 (p = 0.0256, n = 6). A paired two-sided Student’s *t*-test was used to compare the CL and IRI kidneys. Blocks indicate means, while bars indicate the s.e.m. CL = contralateral kidney; HE = hematoxylin and eosin; NGAL = neutrophil gelatinase-associated lipocalin; KIM-1 = kidney injury molecule 1; IRI = ischemia/reperfusion injury.

**Figure 2:**
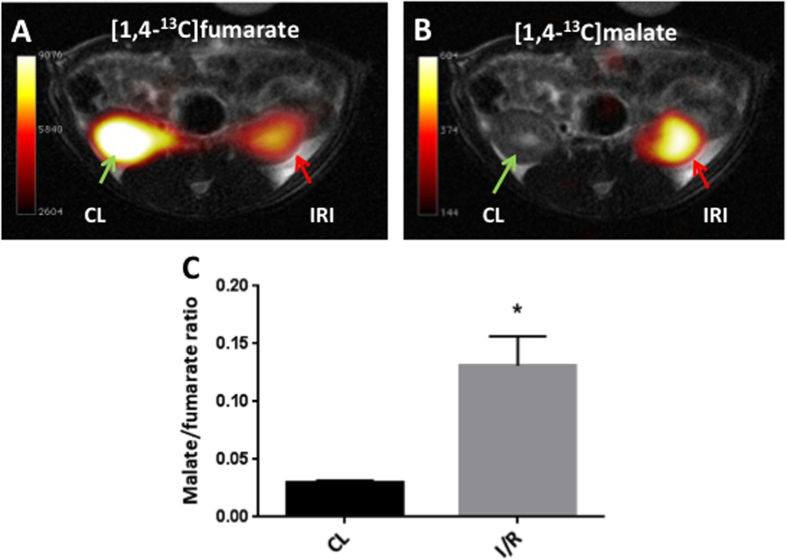
Magnetic resonance imaging maps and malate/fumarate ratios. Representative anatomical ^1^H kidney sections from the post-ischemic animals overlaid with (**A**) ^13^C-labeled fumarate images, and (**B**) ^13^C-labeled malate images. (**C**) A malate/fumarate ratio calculated from each kidney (n = 6 CL, n = 6 IRI), giving rise to an elevated ratio in the post-ischemic kidney (p = 0.0065). The green arrow indicates the CL. The red arrow indicates the IRI kidney. A paired two-sided Student’s *t*-test was used to compare the CL and IRI kidneys. Blocks indicate means, while bars indicate the s.e.m. All relevant abbreviations as in Fig. 1.

**Figure 3:**
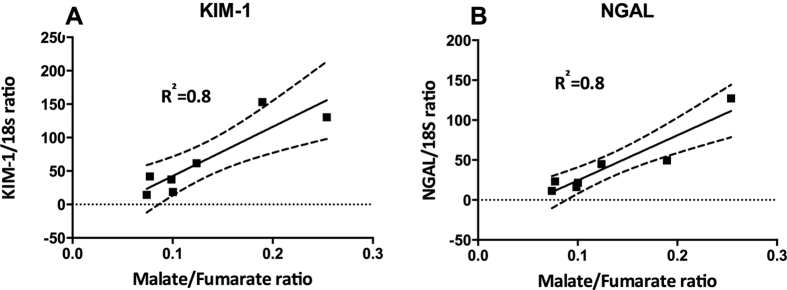
Correlation between renal injury and malate/fumarate ratio. A significant deviation from zero was found between (**A**) NGAL and malate/fumarate ratios (n = 7, p = 0.0017 and R^2^ = 0.88), and (**B**) KIM-1 and malate/fumarate ratio (n = 7, p = 0.0064, R^2^ = 0.80). The dashed line indicates the 95% confidence interval. The straight line indicates the regression line. All qPCR measurements were performed in duplicate. All relevant abbreviations as in Fig. 1. qPCR = quantitative PCR.

**Figure 4:**
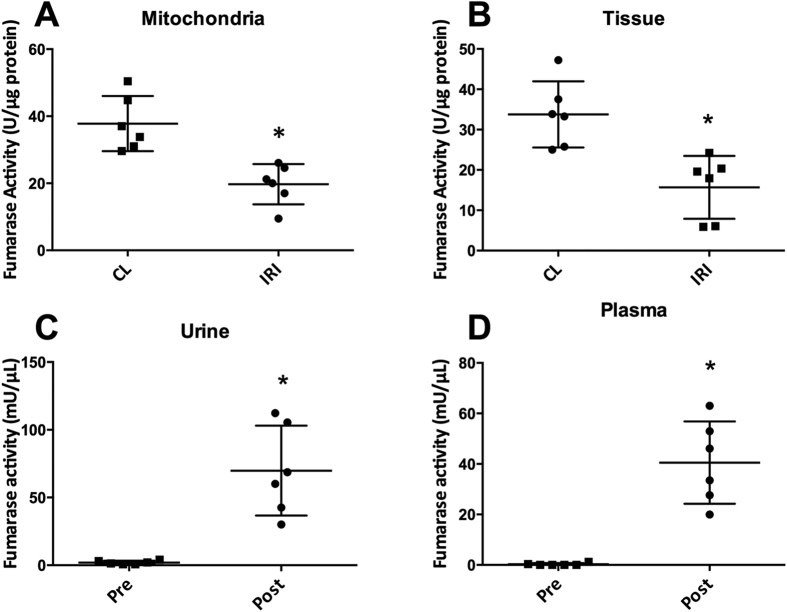
Biochemical analysis of fumarase activity. Fumarase activity was measured in (**A**) the mitochondrial fraction (n = 6, p = 0.0022), (**B**) whole-tissue biopsies (n = 6, p = 0.0067), (**C**) urine (n = 6, p = 0.004), and (**D**) plasma (n = 6, p = 0.0001) isolated from arterial blood samples. A paired two-sided Student’s *t*-test was used to compare the CL and IRI kidneys and the pre and post-surgery urine or plasma samples. All activity measurements were performed in duplicate. Tissue and mitochondrial activity was normalized to protein content, while urine and plasma levels were normalized to the sample volume. Blocks indicate means, while bars indicate the s.e.m. All relevant abbreviations as in Fig. 1.

**Figure 5:**
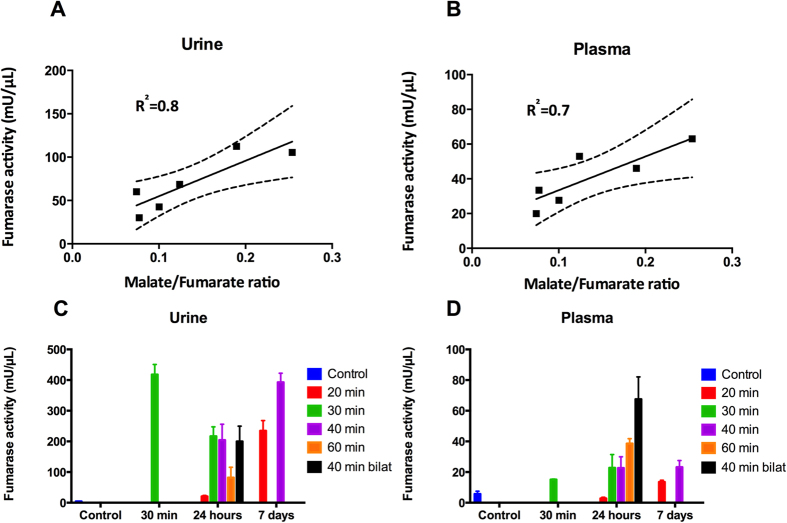
Correlation between urine and plasma fumarase activity and fumarase activity with varying degrees of ischemia/reperfusion injury. A deviation from zero was found between (**A**) Urine fumarase activity and fumarate/malate ratio (n = 6, p = 0.021, R^2^ = 0.77.), and (**B**) Plasma fumarase activity and fumarate/malate ratio (n = 6, p = 0, R^2^ = 0.722). The dashed line indicates the 95% confidence interval. The straight line indicates the regression line. Fumarase activity in 30-min/30-min IRI plasma (p = NS) and urine (p ≤ 0.001), in 20-min/1-day IRI plasma (p = NS) and urine (p = NS), in 40-min/1-day IRI plasma (p = NS) and urine (p = 0.0005), in 20-min/1-week IRI plasma (p = NS) and urine (p = 0.0001), in 40-min/1-week IRI plasma (p = NS) and urine (p ≤ 0.0001), in 40-min/1-day bilateral IRI plasma (p ≤ 0.0001) and urine (p = 0.0006), in 30-min/1-day IRI plasma (p = NS) and urine (p = 0.0003) and in 60-min/1-day IRI plasma (p = 0.0091) and urine (p = NS). In all examples lists, the length of the period of ischemia is given first, followed by that of the period of reperfusion. One-way ANOVA with a Holm-Sidak’s multiple comparisons test was used to compare values between the varying degrees of IRI. All relevant abbreviations as in Fig. 1. ANOVA = analysis of variance; NS = not statistically significant.

A significantly elevated kidney weight (p = 0.001) following 40 min of unilateral ischemia and 24 h of reperfusion was observed. A tendency towards a reduction of body weight of 1.5% ± 4.5% (p = 0.46) and an increase in urine output of 52% ± 70% (p = 0.11) was observed, but did not reach statistical significance. Functional kidney parameters showed consistent signs of renal IRI with an elevated plasma creatinine level of 91% (p = 0.0002) and a reduced CrCl and BUN level of 44% (p = 0.04) and 30% (p = 0.003), respectively, when comparing pre-surgery with post-surgery values (Table 1). Figure 1 shows representative histological sections with hematoxylin and eosin stain from a CL kidney and a post-ischemic kidney. The CL kidney showed normal intact tubular epithelial cells compared to the post-ischemic kidney, with tubular lumina filled with cellular debris, complete sloughing of tubular epithelium, interstitial edema, and glomerular edema (Fig. 1A,B). The classical cortical kidney injury markers NGAL and KIM-1 were significantly elevated (p = 0.01 and p = 0.03) compared to those in the CL kidney (Fig. 1C,D). An elevated malate/fumarate ratio of 339% (p = 0.002) (Fig. 2C) in the ischemic kidneys compared that in the CL kidney was found. In order to examine the relationship between renal cortical injury and malate/fumarate ratio, the correlation between NGAL and KIM-1 levels with the malate/fumarate ratio was investigated. A linear correlation was found in both cases (R^2^ = 0.78, p = 0.008 and R^2^ = 0.80, p = 0.006, respectively) (Fig. 3A,B). To investigate the localization of fumarase in connection with renal IRI, molecular fumarase activity measurements were performed. Fumarase activity in the mitochondrial fraction and the whole tissue was significantly reduced by 48% (p = 0.002) and 54% (p = 0.007) when compared with the values of the CL (Fig. 4A,B). Fumarase activity measured in urine and plasma was significantly elevated (p = 0.004 and p = 0.0001), with practically no activity observed under control conditions (Fig. 4C,D). Fumarase activity measured in urine samples collected immediately after sacrifice was correlated with malate/fumarate ratios (R^2^ = 0.77, p = 0.02) (Fig. 5A), as was plasma fumarase activity (R^2^ = 0.72, p = 0.03) (Fig. 5B). A parallel investigation of fumarase activity in the urine of IRI and control rats showed a time and severity-dependent increase in urine fumarase activity. Elevated activity at as early as 30 min after reperfusion, followed by a reduction 24 h after and another increase in activity after 7 days was observed (Fig. 5C,D). Bilateral IRI was associated with a less pronounced increase in activity in the urine, while the plasma activity was greatly increased (Fig. 5C,D).

## Discussion

The main finding of this study was the significantly elevated hyperpolarized malate/fumarate ratio in response to 40 min of unilateral ischemia and 24 h of reperfusion, and a time and severity-dependent increase in urine and plasma fumarase activity. This elevation correlated with the levels of the well-known renal cortical injury markers KIM-1 and NGAL. Furthermore, the findings verified the original report of Clatworthy *et al*.^33^, who demonstrated that an elevated malate/fumarate ratio could be used as a direct marker of necrosis in renal disease.

All rats included in this study showed evidence of injury in the post-ischemic kidney 24 h after surgery according to the functional kidney parameters plasma creatinine, CrCl, and BUN. Additionally, the histological hematoxylin and eosin-stained sections showed typical signs of tubular necrosis. The molecular markers NGAL and KIM-1 were highly upregulated in the post-ischemic kidney. Although these markers are not specific to necrosis^35^,^36^, they do indicate general injury in the cortical region of the post-ischemic kidney, which in this study was directly correlated with the malate/fumarate ratio. The reduced fumarase activity measured in the post-ischemic kidney compared to that in the CL kidney (whole-tissue and mitochondrial fraction) might seem counter-intuitive, as the malate/fumarate ratio is higher in the post-ischemic kidney. However, as the polarization-relaxation decay of [1,4-^13^C_2_]fumarate is fast compared to the uptake of fumarate through dicarboxylate transporters^37^, malate production is blocked in the CL kidney despite the relatively higher fumarase activity. Meanwhile, in the post-ischemic kidney, the observed signal of hyperpolarized [1,4-^13^C_2_]malate is interpreted as the release of fumarase caused by cellular membrane disruption in connection with necrosis^38^ to the interstitial space, plasma, and urine. Once released from the cells, fumarase, a highly potent enzyme, will produce malate in the presence of fumarate. This is caused by an equilibrium constant favoring malate formation (K_c_ = 4.3, pH 7.5)^39^, but also the fact that fumarase requires no co-substrates or co-enzymes to function, meaning that even though fumarase is exclusively intracellular, it functions just as well in the extracellular space under necrotic conditions.

**Figure 6:**
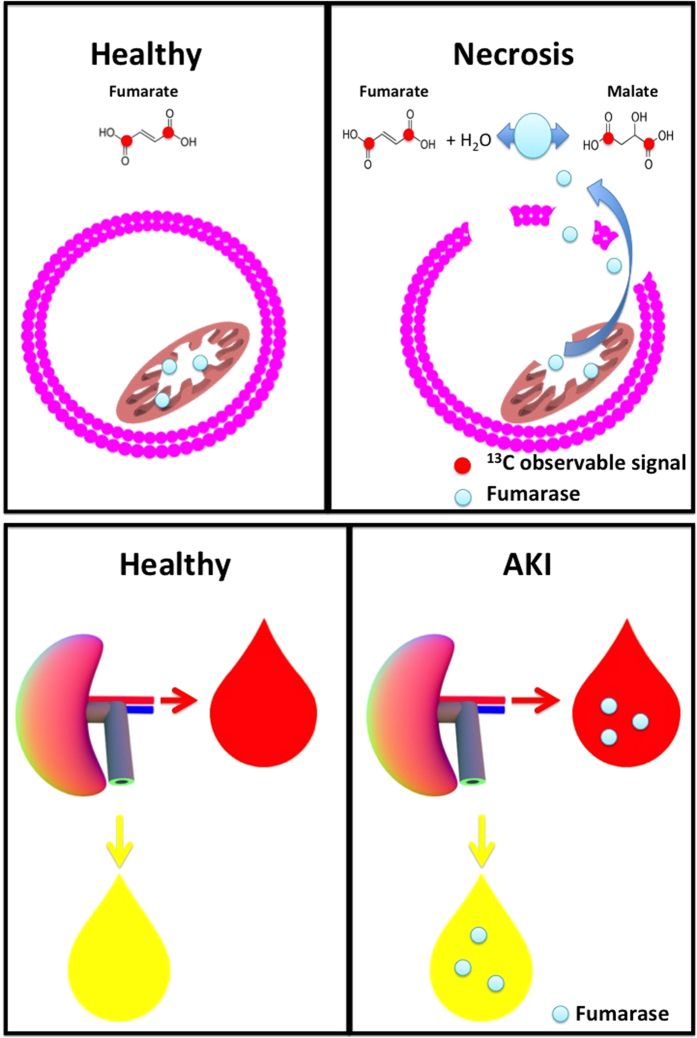
Proposed hypothesis for the sensitive biomarker of renal necrosis in AKI. In healthy cells the transport of hyperpolarized [1,4-^13^C]fumarate across the cell membrane is slow compared to the decay of the hyperpolarized signal and thus no conversion to [1,4-^13^C]malate via fumarase is seen. In necrotic cells the plasma membrane is compromised and thus fumarase is freely available for the substrates [1,4-^13^C]fumarate and water to convert to [1,4-^13^C]malate. Additionally in the healthy rat little or no fumarase are present in blood or urine, however following AKI both blood and urine show necrosis dependent fumarase activity.

Plasma and urine fumarase activity levels were highly increased after the onset of IRI, and were correlated with the hyperpolarized malate/fumarate ratios. Albeit giving rise to measurements characteristic of IRI, these values, like other blood/urine biomarkers of AKI, were unable to specify which kidney (or both) was suffering from necrotic injury. Several complementary MRI techniques have previously demonstrated promising results for AKI monitoring. Conventional perfusion imaging using either arterial spin labeling or contrast agents, have demonstrated reduced perfusion in the post-ischemic kidney^40^,^41^, similarly recently found with hyperpolarized ^13^C-urea^42^. Diffusion weighted imaging (DWI), a well-suited imaging marker of renal complications have shown diffusion restrictions already at onset of AKI and this restriction is associate with the severity of AKI, inflammatory cell infiltration and interstitial renal fibrosis^43^. Relaxation mechanisms has been shown to be related to edema, fibrosis and renal (oxygenation blood-oxygen-level-dependent contrast imaging (BOLD))^43^,^44^,^45^. The ability to non-invasively monitor both anatomical, hemodynamic, metabolic renal changes associated with AKI with sufficient sensitivity and specificity, support the use of MRI in both animal and patients studies. The addition of hyperpolarized [1,4-^13^C]fumarate for local necrosis examinations to this powerful MR toolbox, further improves this toolbox. The combination of urine and plasma fumarase measurement with hyperpolarized MRI procedures shows great promise for future clinical translation. Figure 6 illustrates the proposed mechanisms for the new sensitive biomarker of renal necrosis in AKI. Furthermore, in some cases, the animal’s fluid balance and fluid intake after IRI induction are associated with a high degree of variation, which will inevitably be reflected in the levels of plasma and urine fumarase activity. Interestingly, the bilateral IRI model showed comparable fumarase activity in the urine compared to that in the unilateral kidneys. While the plasma activity was significantly elevated, this is most likely due to a reduced urine output in the bilateral cases. The same situation was seen in the 60-min IRI model, wherein the post-ischemic animals had very little urine output, leading to very little secretion of fumarase. Therefore, enzymes released from necrotic tissue are mainly seen in the circulation (plasma fraction). In the other IRI cases, there was elevated urine output, which is often seen in early/moderate IRI cases^46^. Therefore, we conclude that fumarase is mainly found in the urine.

In conclusion, these results highlight the potential for following disease progression using a simple urine and plasma test and the verification of the degree and location of the damage with more sensitive imaging tests such as MRI. We demonstrated a correlation between renal necrotic injury in connection with IRI with *in vivo* and *in vitro* fumarase activity, and described the underlying mechanisms of the proposed methods. We believe that the simple measurement of fumarase activity measurement in the blood and urine, in combination with hyperpolarized MRI, holds great promise as a future diagnostic tool for AKI. These findings should serve as a starting point for future research in human subjects.

## Methods

### Animal models

The imaging experiments were performed on male Wistar rats (weighing 200–250 g). The animals were provided with ad libitum access to a standard rodent diet (Altromin, Germany) and tap water, and were kept under a 12/12-h light-dark cycle at a temperature of 21 ± 2 °C and a humidity of 55 ± 5%. The studies were carried out in accordance with the Danish National Guidelines for animal care, and were approved by the Danish Animal Experiments Inspectorate under the Danish Veterinary and Food Administration (License no. 2013/15-2934-00810).

During surgery (animal order randomized), the animals were placed on a heating pad (CMA 450 temperature controller, Harvard apparatus) to maintain a rectal temperature of approximately 36–37 °C, and respiration was visually monitored. A surgical incision was made in the abdomen, and the left renal artery was carefully dissected. A non-traumatic clamp was placed on the left artery for 40 min to induce ischemia, after which the clamp was released. Reperfusion was visually confirmed. The incision was sutured separately through both the muscle tissue and skin. The contralateral (CL) kidney was left intact, and was used as the control kidney. During surgery, the animals were anesthetized with sevoflurane (induction 6%, sustained 2.5%) mixed with air (2 L/min). At the beginning of surgery, Temgesic (buprenorphine hydrochloride) sublingual tablets were provided subcutaneously (0.05 mg/kg), after which buprenorphine hydrochloride was supplied in the drinking water (0.3 mg/mL) until euthanization. To maintain post-operative water balance, 2 mL of isotonic salt water was injected subcutaneously at the beginning of the operation. Prior to surgery, the rats were kept in metabolic cages. After 24 h in the metabolic cage, urine was collected and the rats were anesthetized for blood sample collection and surgery. After surgery, the rats were again put in metabolic cages. At the time of euthanization (24 h after surgery), arterial blood and urine was collected again to estimate fumarase activity.

A total of seven animals were used for the imaging experiments. From six of these animals, urine and blood samples were successfully extracted and used to measure fumarase activity (unsuccessful blood sampling lead to the loss of blood and urine measurements from one animal). The same seven animals were then used for quantitative polymerase chain reaction (qPCR) measurements of tissue and mitochondrial fumarase activity following 40 min of unilateral ischemia and 24 h of reperfusion.

Urine and plasma sampling was performed on a total of 42 animals. The animals were placed in groups of six, and varying degrees of IRI were then induced. One group was exposed to 30 min of ischemia and 30 min of reperfusion. Two groups were exposed to 20 min and 40 min of ischemia, and urine and plasma samples were collected after 24 h and 1 week of reperfusion. Two groups were exposed to 30 min and 60 min of ischemia and 24 h of reperfusion. One group was exposed to 40 min of bilateral ischemia followed by 24 h of reperfusion. Finally, a sham-operated group was included to serve as a control.

### Renal histology

A 2-mm kidney section was dissected from both the CL kidney and post-ischemic kidney from each rat at the time of euthanasia. The kidney sections were fixed in 4% paraformaldehyde for 2 h and washed 3 times (10 min) with 0.01 M phosphate-buffered saline. The fixed kidneys were then dehydrated, embedded in paraffin, and cut into 2-μm sections on a rotary microtome (Leica Microsystems A/S, Herlev, Denmark). The paraffin-embedded sections were stained with hematoxylin and eosin to evaluate the presence of tubular necrosis. Evaluation was performed blinded under high magnification (20x). Representative images are shown at 20x magnification.

### Activity assays

Fumarase activity was measured in plasma, urine, whole renal cortex tissue, and mitochondrial fractions according to the manufacturer’s instructions (Sigma Aldrich, Brøndby, Denmark). Fumarase activity in the mitochondria and tissue was normalized to the amount of protein in the sample. Plasma and urine fumarase activity was normalized to the amount of sample added to the assay. The mitochondrial fraction was isolated using Dounce homogenization of freshly dissected renal tissue followed by several centrifugal steps. Mitochondrial purity was verified by Western blotting. The tissue and mitochondrial fractions were then homogenized in the fumarase assay buffer. Analysis was performed in 96-well costar half plates using a PHERAstar FS micro plate reader (BMG Labtech, Birkerød, Denmark). Urine and/or plasma were distributed without pre-treatment in 96-well costar half plates. Fumarase activity in urine was assessed at a wavelength of 670 nm instead of the usual 650 nm because of the presence of background interference.

### RNA extraction and quantitative PCR

Total RNA was isolated from the renal cortex using a NucleoSpin RNA II mini kit according to the manufacturer’s instructions (AH diagnostics, Aarhus, Denmark). RNA was quantified by spectrophotometry and stored at −80 °C. cDNA synthesis was performed with a RevertAid First strand cDNA synthesis kit (MBI Fermentas, Burlington, Canada). qPCR was performed using Maxima SYBR Green qPCR Master Mix according to the manufacturer’s instructions (AH diagnostics, Aarhus, Denmark). Briefly, 100 ng of cDNA was used as a template for PCR amplification. The specificity of products was confirmed by melting curve analysis and gel electrophoresis. Primer sequences used included: 18 s forward 5′-CAT GGC CGT TCT TAG TTG-3′ and reverse 5′-CAT GCC AGA GTC TCG TTC-3′ designed from ascension no: M11188; KIM-1 forward 5′-CCA CAA GGC CCA CAA CTA TT-3′, and reverse 5′-TGT CAC AGT GCC ATT CCA GT-3′ designed from ascension no: AF035963; and NGAL forward 5′-GAT CAG AAC ATT CGT TCC AA-3′ and reverse 5′-TTG CAC ATC GTA GCT CTG TA-3′ designed from ascension no: BC089053.

### Hyperpolarized experiments

At the MRI scanning session 24 h after IRI surgery, tail vein catheterization was performed for hyperpolarized [1,4-^13^C_2_]fumarate administration. The animals were placed in a clinical 3 T MRI scanner (GE Healthcare, Waukesha, US) for imaging. Throughout the experiment, the animals were anesthetized with sevoflurane (2.5% sevoflurane and 2 L/min air). Rectal temperature, pO_2,_ and respiration were monitored throughout the MRI session. Each animal was injected with 1.5 mL of hyperpolarized 30 mM [1,4-^13^C_2_]fumarate. The pH was 7.4 and the solution was isotonic.

The [1,4-^13^C_2_]fumarate was polarized in a SPINlab based on Ardenkjaer-Larsen *et al*.’s original polarizer design^47^. (GE Healthcare, Brøndby, Denmark). The [1,4-^13^C_2_]fumarate sample was prepared by dissolving [1,4-^13^C_2_]fumaric acid (FA) (Cambridge Isotopes, Cambridge, UK) to a final concentration of 3.6 M in dimethyl sulfoxide containing the trityl radical (12 mM AH111501, GE Healthcare, Brøndby, Denmark). The fluid path was prepared by placing 100 μL (about 350 μmol) of the FA solution in the sample cup and then freezing it in liquid nitrogen. The remainder of the fluid path preparation was performed according to the manufacturer’s instructions. The FA solution was allowed to melt for 10–30 min before lowering the fluid path into the helium bath. The sample vial was lowered in a fast two-step scheme to avoid the crystallization of the FA in the sample. The sample was polarized for approximately 3 h to a reproducible polarization of approximately 40%. The dissolution syringe was filled with approximately 15 g of a dissolution media (sterile water with 0.1 g/L EDTA). After dissolution, the sample was mixed with 0.54 g of neutralizing buffer (sterile water with 0.72 M NaOH, 0.4 M TRIS, and 0.1 g/L EDTA).

MRI scans were performed using a 3 T clinical MRI system (GE Healthcare, Brøndby, Denmark) equipped with a dual tuned ^13^C/^1^H volume rat coil (GE Healthcare, Brøndby, Denmark). A slice-selective ^13^C IDEAL spiral sequence was used to detect hyperpolarized [1,4-^13^C_2_]fumarate, and images were acquired every 5 s, initiated 20 s after the start of injection. The spiral acquisition was performed using a flip angle of 10°, 11 IDEAL echoes, and one initial spectrum per IDEAL encoding^48^. The following parameters were also used: TR/TE/ΔTE = 100 ms/0.9 ms/1.45 ms; field of view = 80 × 80 mm^2^; 5 × 5 mm resolution interpolated to a 0.3-mm resolution; and an axial slice thickness of 15 mm covering both kidneys. The ^13^C/^1^H images were converted to the DICOM format and analyzed using Osirix software^49^. Images of [1,4-^13^C_2_]fumarate and [1,4-^13^C_2_]malate were overlaid on anatomical ^1^H images: representative images are provided in Fig. 3A,B. Analysis was performed according to the region of interest (ROI). The ROIs were placed around each kidney on the ^1^H images and transferred to the ^13^C images. The area under the time curve ratio between the hyperpolarized [1,4-^13^C_2_]malate signal and the hyperpolarized [1,4-^13^C_2_]fumarate signal from each individual kidney was calculated^50^.

### Statistics

All data are presented as means ± s.e.m. Normality was assessed with quantile plots. A P-value < 0.05 was considered statistically significant. A paired Student’s *t*-test was used to compare values between the CL kidney and the post-ischemic kidney. The linear correlation was tested between the kidney injury markers Kim-1, NGAL, and the corresponding malate/fumarate ratio.

One-way analysis of variance with a Holm-Sidak’s multiple comparisons test was used to evaluate fumarate activity in the urine and blood collected from animals with varying degrees of IRI. A linear regression test was performed on the qPCR measurements of KIM-1 and NGAL, which were tested against the corresponding malate/fumarate ratios. The goodness of fit was calculated to provide R^2^ values, and the deviation from zero was also calculated. Statistical analyses were performed using GraphPad PRISM 6.

## Additional Information

**How to cite this article**: Nielsen, P. M. *et al*. Fumarase activity: an *in vivo* and *in vitro* biomarker for acute kidney injury. *Sci. Rep.* **7**, 40812; doi: 10.1038/srep40812 (2017).

**Publisher's note:** Springer Nature remains neutral with regard to jurisdictional claims in published maps and institutional affiliations.

## Figures and Tables

**Table 1:** Renal function parameters before and after surgery.

	Body weight (g)	Kidney weight (mg/g bodyweight)	Urine output (μL/min/kg)	Plasma creatinine (μmol/L)	CrCl (mL/min/kg)	BUN (μL/min/kg)
Pre Surgery (n = 6)	247 ± 6.3	—	30.7 ± 9.4	15 ± 1.7	10.1 ± 1.5	4.9 ± 0.7
Post Surgery (n = 6)	243 ± 9.5 (NS)	IRI 4.5 ± 0.1* CL 3.7 ± 0.04	41.6 ± 8.5 ns.	28.7 ± 2.4*	5.7 ± 3.2*	7.0 ± 0.5*

CL = contralateral kidney; IRI = ischemia/reperfusion injury; CrCl = creatinine clearance; BUN = blood urea nitrogen; NS = not statistically significant. Values are given as mean ± s.e.m. *Indicate significant difference of P-value < 0.05.
